# A novel approach to utilizing the essential public health functions in Ireland's health system recovery and reform

**DOI:** 10.3389/fpubh.2023.1074356

**Published:** 2023-03-03

**Authors:** Triona McNicholas, Louise Hendrick, Geraldine McDarby, Saqif Mustafa, Yu Zhang, Sohel Saikat, Zsuzsanna Jakab, Tony Holohan

**Affiliations:** ^1^Department of Health, Dublin, Ireland; ^2^The World Health Organization, Geneva, Switzerland

**Keywords:** public health, COVID-19, health system resilience, lessons learned, essential public health functions

## Abstract

This article is part of the Research Topic ‘Health Systems Recovery in the Context of COVID-19 and Protracted Conflict.’

The COVID-19 pandemic presented a challenge to health systems and exposed weaknesses in public health capacities globally. As Ireland looks to recovery, strengthening public health capacities to support health systems resilience has been identified as a priority. The Essential Public Health Functions (EPHFs) provide an integrated approach to health systems strengthening with allied sectors and their operationalization supports health systems and multi-sectoral engagement to meet population needs and anticipate evolving demands. The Health Systems Resilience team (World Health Organization, HQ) in collaboration with the Department of Health (Ireland) developed a novel approach to the assessment of the EPHFs in Ireland. The approach involved a strategic and focused review of the delivery and consideration of EPHFs in relation to policy and planning, infrastructure, service delivery, coordination and integration, monitoring and evaluation and learning. Informed by a literature review and key document search, key stakeholder mapping and key informant interviews, lessons learned from experience with COVID-19 nationally and internationally, strengths as well as potential areas of improvement to optimize delivery of EPHFs were identified. Mapping of the EPHFs in Ireland revealed that there is evidence of delivery of all 12 EPHFs to varying degrees; however a number of challenges were identified, as well as numerous strengths and opportunities. Recommendations to optimize the delivery of EPHFs in Ireland include to integrate and coordinate EPHFs, increase the visibility of the public health agenda, leverage existing mechanisms, recognize and develop the workforce, and address issues with the Health Information System. There is a public health reform process currently underway in Ireland, with some of these recommendations already being addressed. The findings of this process can help further inform and support the reform process. Given the current focus on strengthening public health capacities globally, the findings in Ireland have applicability and relevance in other WHO regions and member states for health systems recovery and building back better, fairer and more resilient health systems.

## 1. Introduction

The COVID-19 pandemic presented an unprecedented challenge to health systems globally and has highlighted the importance of building health system resilience. A resilient health system can effectively prevent, prepare for, respond to, and adapt to public health challenges while maintaining routine health system functions (1). Despite numerous warnings from public health officials, infectious disease experts and previous international commissions and reviews, the world was not prepared to respond to the COVID-19 pandemic (2). This, in addition to numerous other public health emergencies (PHE) such as the SARS epidemic in 2003, the H1N1 influenza pandemic in 2009, the Ebola outbreak in West Africa in 2014–2016, Zika, MERS, and other threats have demonstrated insufficiencies in actions to build health systems resilience globally. The World Health Organization (WHO) has recognized the essential public health functions (EPHFs) as a key strategy to build health system resilience and has called on countries to strengthen EPHFs and health systems foundations (3, 4).

The EPHFs are a fundamental set of collective actions under the primary responsibility of the state, which help to ensure effective public health actions, including the protection, maintenance and promotion of population health (4). They can be regarded as the capabilities that health authorities, in collaboration with other relevant sectors should build and strengthen within health and allied systems, and they are key to ensuring a holistic approach to public health from policy and planning to the provision of services (4, 5). Many countries, WHO regions and partners have developed EPHF frameworks that reflect their priorities and contexts. Although differences exist in these frameworks, there are significant commonalities such as a focus on health promotion, prevention, protection, and actions on the wider determinants of health and equity. The WHO recently developed a consolidated set of EPHFs and has proposed this as a reference list of activities for countries to ensure effective public health action for acute threats, evolving challenges and chronic stressors including the COVID-19 pandemic (4). The EPHFs advocate for proportionate investment in public health in relation to costly secondary and tertiary care and provide an integrated approach to health system strengthening.

When assessed in terms of average case rates, deaths and excess mortality, Ireland performed relatively well in response to the COVID-19 pandemic compared to many European countries (6). However, the pandemic response that was required to achieve this has had a substantial impact on essential health service delivery, including pausing of screening programmes during wave 1, reduced GP attendance, and disruption to elective care (7). Additionally, the direct effects of COVID-19, including long COVID-19, and the indirect consequences such as functional and cognitive decline, loneliness, low mood, anxiety, alcohol dependence, and weight gain are likely to have an impact on the health system into the future (7). Ireland currently has a relatively young population compared to the rest of Europe, however the demographic profile in Ireland is changing, placing substantial and sustained pressure on health services. Unlike most European countries, the size of the population in Ireland is increasing. The preliminary results from the most recent census (2022) demonstrated that the population has grown by 7.6% since the last census (2016) to 5.1 million (8). This population growth is projected to continue for at least the next two decades, with increases in the older age groups projected to continue quickly and steeply (9). Older age cohorts are the highest users of the majority of health care services and increases in these age cohorts will have a significant impact on demands for health services in the future and their integrated delivery (9). Prior to the pandemic, Ireland's health system was already under strain due to longstanding weaknesses, with long waiting times to access health services and diagnostics (10, 11), and the changing demographics of the Irish population are likely to exacerbate problems if steps are not taken to bolster preventative health and health protection capacities and build health system resilience.

The Department of Health (DoH) provides governance of health and social care services. The Health Service Executive (HSE) is responsible for delivery of services and implementation of initiatives set out in the annual National Service Plan (NSP). In 2019, governance structures were strengthened with regular high-level meetings between both organizations and introduction into law of the HSE Governance Act (2019) which formally established a new HSE board accountable to the minister and led to appointment of a Chief Executive Officer (CEO) accountable to the board (12). There is broad consensus that the current health system in Ireland is overly hospital-centric, with community-based services that are fragmented with a lack of integration of care within and across different services (9). Reactive care takes precedence over proactive and preventative care and the system in its current form is not meeting the needs of patients. Therefore, the health system is undergoing a period of transformation and reform with the implementation of a 10-year cross party and cross governmental plan for transformation of the Irish health system published in 2017, “Sláintecare” (13). Sláintecare seeks to deliver universal access to high quality health services based on the reorientation of the system toward integrated primary health and community care. Although implementation has been modest to date and complicated by the COVID-19 pandemic, reform is ongoing and presents an opportunity to embed public health and a population-based approach to healthcare into a reformed system.

Responsibility for Public Health has historically been situated within the Health and Wellbeing division of the HSE, although its functions are scattered throughout the HSE. A statutory public health function in the role of “Medical Officer of Health” (MOH) is established in Ireland under the 1947 and 1953 Health Acts, and the Infectious Disease regulations 1981 (and subsequent amendments). A new model for the delivery of public health was developed in 2019 following the recommendations of an independent review, which recommended the development of a ‘hub and spoke' model of service delivery, encompassing all domains of public health practice that enables strong public health leadership supported by multidisciplinary teams (14). Recruitment into this new model is currently underway.

The Health Systems Resilience team (World Health Organization, HQ) in collaboration with the Department of Health (Ireland) developed a novel approach to the assessment of the EPHFs in Ireland. The purpose of this assessment was to present an overview of the current delivery of the EPHFs to inform national policy for building health systems resilience as the country recovers from the COVID-19 pandemic.

## 2. Methods

In order to align with the timeline of a governmental reform process within Ireland, a rapid approach to the assessment of EPHF delivery was developed. The consolidated list of 12 EPHFs proposed as a reference for countries by WHO was reviewed by the joint team with agreement on definition, components and scope of each EPHF (Table 1) (4). A document search including academic literature, gray literature and government policy documents was then conducted to inform the current delivery and consideration for EPHFs as assessed across four key pillars; policy and planning, inputs and infrastructure, service delivery and coordination and integration. Two cross cutting areas; monitoring and evaluation and learning systems were also examined (Supplementary material). Documents were assessed using a key questions matrix developed by the joint working team (Supplementary material). Findings were then crosschecked and triangulated using key informant interviews, with interviewees identified through stakeholder mapping of EPHF delivery (Supplementary material).

**Table 1:** Fundamental list of essential public health functions.

1. Monitoring and evaluating the population's health status, health service utilization and surveillance of risk factors and threats to health
2. Public health emergency management
3. Assuring effective public health governance, regulation, and legislation
4. Supporting efficient and effective health systems and multisectoral planning, financing, and management for population health
5. Protecting populations against health threats, including environment and occupational hazards, communicable disease threats, food safety, chemical and radiation hazards
6. Promoting prevention and early detection of diseases, including noncommunicable and communicable diseases
7. Promoting health and well-being and actions to address the wider determinants of health and inequity
8. Ensuring community engagement, participation, and social mobilization for health and well-being
9. Ensuring adequate quantity and quality of public health workforce
10. Assuring quality of and access to health services
11. Advancing public health research
12. Ensuring equitable access to and rational use of essential medicines and other health technologies

## 3. Results

### 3.1. Mapping of the delivery of EPHFs in Ireland

**Figure 1:**
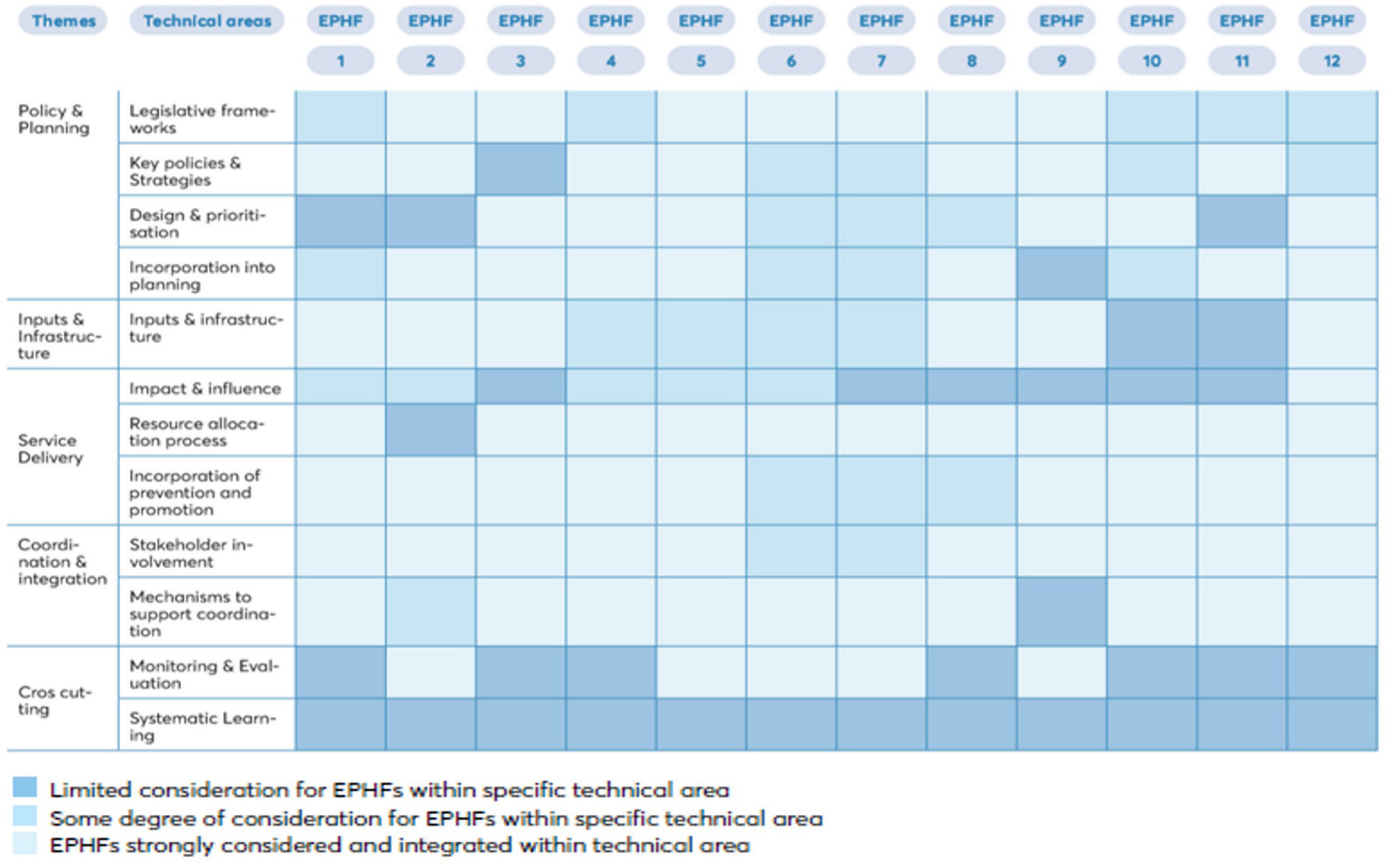
Visual representation of mapping of individual EPHFs against key technical areas.

The agreed list of 12 fundamental EPHFs (Table 1) formed the basis of the mapping process. A visual representation of mapping of individual EPHFs against key technical areas is provided in Figure 1. A number of the main findings are listed below.

- There is evidence of delivery of all 12 EPHFs to varying degrees; however, there is limited evidence of a coordinated approach, with some EPHFs being delivered directly through the health system and others in partnership with public bodies, non-governmental organizations, academia and other sectors.
- There is limited evidence of an overarching strategy, policy or governance structure to coordinate the planning and delivery of EPHFs across the system.
- Delivery of the EPHFs is siloed with respect to strategy, planning, financing, implementation and monitoring and evaluation mechanisms, and there appears to be limited consideration given to health system strengthening or identifying opportunities for synergies. This can contribute to duplication, gaps and inefficiencies.
- There are examples of a more coordinated approach such as Healthy Ireland structures that seek to integrate prevention and health promotion.There is no overarching governance structure for the delivery of EPHFs, and governance structures vary from national structures, national to regional structures, or regional only structures. A number of vertical programmes report into the Office of the Chief Clinical Officer on the Executive Leadership board of the Health Service Executive (HSE), the agency responsible for the delivery of health and social care services in Ireland, while others report through the office of strategic planning in the HSE. Legislation is in place to support many of the threats defined in EPHF5 (health protection) and EPHF10 (quality and access). However, legislation to support the delivery of all EPHFs is limited and what is in place applies to control of infectious diseases and is not specific to emergency response, which led to the need for a substantive amount of drafting of primary legislation in response to COVID-19.
- There are strong emergency focused inter-sectoral mechanisms and mechanisms that support inter-sectoral and international collaboration and information sharing, however at an operational level there is no lead agency mandated and resourced to lead emergency preparedness and response. The DOH is the lead government agency responsible for pandemic planning and health security structures, and the HSE is responsible for operational delivery of the health system's pandemic response.
- The Health Protection Surveillance Center (HPSC) within the HSE serves as the International Health Regulations focal point; however, it does not have the capacity to be the lead agency in preparedness and response within the HSE. There is evidence of a mismatch between the scope of public health activities outlined within national strategies and policies, and what is supported by planning, with a focus on health protection in terms of resourcing and infrastructure. The National Service Plan, which sets out the types and volume of health and social services to be provided on an annual basis, outlines resources for health protection (EPHF5) only.
- Although a significant amount of data informs health systems planning, operational limitations exist in the health information systems and infrastructure. Health information infrastructure is fragmented, with multiple data collection points and data repositories, with a lack of clarity around data access and sharing, and limited integration and linkage between systems. A dearth of modeling capacity in the HPSC existed prior to the COVID-19 pandemic.

### 3.2. Strengths and opportunities identified

Despite these challenges, the Irish health system performs relatively well across many health and health systems indicators, including in the context of the COVID-19 pandemic. However, Ireland is now entering a challenging time in the recovery period, and is facing increasing pressures on health systems with demographic changes and a backlog of demand stemming from the pandemic. Although this leaves the health system vulnerable to ongoing and future public health emergencies, there were a number of strengths identified that can be leveraged to build health systems resilience:

1. There is considerable capacity to deliver EPHFs within the system;
2. There is a high level of public health expertise within the system;
3. The Irish health workforce is resourceful and agile;
4. There is substantial evidence generation and synthesis capacity within the system;
5. There is a recognition of the need for a whole of society, whole of government approach to health;
6. Data informed planning is evident at a high level within the heath sector, despite infrastructural and capacity issues identified.

### 3.3. Potential areas for improvement

This review aimed to identify actions that could address gaps and optimize delivery of EPHFs in Ireland. Several opportunities were identified that could support optimal delivery of the EPHFs. These include:

**1. Integrate and coordinate EPHFs to reduce fragmentation and promote efficiency and effectiveness. This can be achieved by:**

- Utilizing EPHFs to define the scope of public health.
- Developing a national public health strategy, provide appropriate financing mechanisms and ensure existing capacity is leveraged.
- Developing Key Performance Indicators for EPHFs and health system resilience, relevant to population health outcomes at national and subnational levels.

**2. Increase the visibility and profile of the public health agenda in the Irish setting. This can be achieved by:**

- Identifying the appropriate strategic placement and resourcing of a coordinating structure for public health.
- Reviewing the governance structures for the delivery of the EPHFs.
- Reviewing institutional arrangements for the delivery of public health at all levels.

**3. Sustain and leverage existing mechanisms in support of a whole-of-government and whole-of-society approach to health, including emergency preparedness and response. This can be achieved by:**

- Defining the new baseline for national systems, taking account of the additional resources and structures within the current, COVID-19 focused baseline.
- Identifying the structures and coordinating platforms to be sustained and leveraged to support an integrated whole-of-government approach to health.
- Sustaining and harnessing the existing mechanisms promoting whole-of-society participation in health.

**4. Define, recognize and develop the public health workforce to ensure that it is capable of adapting to ongoing and evolving public health challenges. This can be achieved by:**

- Defining the skill set and competencies of the public health workforce required to effectively deliver the EPHFs.
- Profiling and mapping the wider public health workforce and develop appropriate mechanisms to enable surge capacity during public health emergencies.
- Developing national and regional strategies for addressing priority gaps in workforce.

**5. Address critical Health Information System issues to ensure appropriate and timely data is available to effectively respond to all public health challenges. This can be achieved by:**

- Reviewing the ICT strategy to ensure recognized ICT issues with respect to infrastructure, security and digitalization are addressed and resourced.
- Ensuring integration and interoperability of data and systems across and between health and allied sectors.
- Ensuring sustainable modeling capacity, evidence synthesis and public health intelligence are in place.
- Ensuring the Health Information Bill recognizes the need for public health intelligence as distinct from health system performance data.

## 4. Discussion

The approach taken in this collaboration identified lessons learned internationally from the COVID-19 pandemic and their relevance to the Irish context, mapped the current delivery of the EPHFs in the Health System in Ireland, presented strengths and opportunities that can be leveraged to enhance the effectiveness of recovery efforts and build health systems resilience and identified actions to address gaps and optimize the delivery of public health in Ireland at a national and subnational level.

While the pandemic is ongoing and it remains too early to draw definite lessons from the pandemic response, lessons identified at this stage can inform the context for strengthening public health capacity in Ireland. This collaborative review has identified numerous strengths and opportunities within the Irish health system and presented recommendations for optimizing delivery of the EPHFs in Ireland, grounded within the national context as well as informed by experience with COVID-19. The capacity to deliver the EPHFs is readily identifiable within the Irish health system. Some integration is apparent, although mainly *ad-hoc* and informal, and this increased during the COVID-19 pandemic. This presents an opportunity to harness existing capacity and maximize synergies across EPHFs and align with current and evolving population health needs. Optimization of delivery of the EPHFs can help to ensure comprehensive and integrated delivery of public health services in Ireland following the COVID-19 pandemic, in response to the health needs of the population with a specific focus on vulnerable groups (e.g., elderly and migrants), and in anticipation of evolving disease profiles (e.g., multimorbidity). The findings of the review are being utilized to support high level advocacy for the strategic shift toward public health required to build and ensure health system resilience against future threats through the EPHFs.

Operationalizing the EPHFs in Ireland can help ensure public health challenges are met affordably and sustainably. As COVID-19 and past and ongoing public health stressors have demonstrated, the cost of inaction, and an overreliance on reactive secondary and tertiary healthcare, is too high. There is an urgent need for adequate and proportionate attention and investments in building preventative, health protective and health promotive capacities utilizing EPHFs for high level planning and advocacy within and beyond the health sector. Implementation of multi-sectoral approaches and health in all policies are also needed given the likelihood of future health systems shocks and stressors, be it from infectious disease outbreaks, climate related events, antimicrobial resistance or rising rates of non-communicable diseases and mental health conditions.

There are a number of limitations to this review. The timeline for analysis was short, in order to align with the ongoing public health reform process in Ireland and leverage political interest and support, limiting the options in terms of approach and granularity of findings. The timeframe informed the study design, and the review is not as in depth as previous assessments of EPHF. However, the review provided concrete and actionable policy options to optimize delivery of EPHFs in the Irish setting. The constantly evolving structures in response to the COVID-19 pandemic and the ongoing health sector reforms created a challenge in understanding the up-to-date delivery of EPHFs in Ireland. The majority of documents reflected delivery of EPHFs prior to the COVID-19 pandemic with some related to the pandemic response and other reflecting changing priorities during the pandemic. Some recent changes in EPHFs may not have been captured during this process. Key informant interviews addressed this challenge to some extent.

## 5. Conclusion

Building resilience into a reformed health system will be key in ensuring Ireland's ability to respond to future threats such as pandemics. Operationalization of the EPHFs can help ensure the health system is prepared to meet the next challenge affordably and sustainably. The findings of the review have been utilized to support high level advocacy for the shift toward public health required to build and ensure health system resilience against future threats. Given the current focus on strengthening public health capacities globally, the findings in Ireland have applicability and relevance to policy audiences and key decision makers within Ireland as well as more broadly to other WHO regions and member states for health systems recovery and building back better, fairer and more resilient health systems.

## Data availability statement

The original contributions presented in the study are included in the article/Supplementary material, further inquiries can be directed to the corresponding author.

## Author contributions

TM: analysis and interpretation of results and draft manuscript preparation. LH: analysis and interpretation of results. GM, SM, and YZ: data collection and analysis and interpretation of results. SS: analysis and interpretation of results and study conception and design. ZJ and TH: study conception and design. All authors contributed to the article and approved the submitted version.
