# Corrigendum: A novel approach to utilizing the essential public health functions in Ireland's health system recovery and reform

**DOI:** 10.3389/fpubh.2023.1187990

**Published:** 2023-04-18

**Authors:** Triona McNicholas, Louise Hendrick, Geraldine McDarby, Saqif Mustafa, Yu Zhang, Sohel Saikat, Zsuzsanna Jakab, Tony Holohan

**Affiliations:** ^1^Department of Health, Dublin, Ireland; ^2^The World Health Organization, Geneva, Switzerland

**Keywords:** public health, COVID-19, health system resilience, lessons learned, essential public health functions

In the published article, there was an error. A correction has been made to the Conclusion section.

The Conclusion previously stated:

“Building resilience into a reformed health system will be key in ensuring Ireland's ability to respond to future threats such as pandemics. Operationalization of the EPHFs can help ensure the health system is prepared to meet the next challenge affordably and sustainably. The findings of the review have been utilized to support high level advocacy for the shift toward public health required to build and ensure health system resilience against future threats. Work is currently underway to utilize the EPHFs to define the operational scope of public health in Ireland and to identify the scope and functions of a new national public health institute and will help inform ongoing implementation of public health reform. Given the current focus on strengthening public health capacities globally, the findings in Ireland have applicability and relevance to policy audiences and key decision makers within Ireland as well as more broadly to other WHO regions and member states for health systems recovery and building back better, fairer and more resilient health systems.”

The corrected section appears below:

“Building resilience into a reformed health system will be key in ensuring Ireland's ability to respond to future threats such as pandemics. Operationalization of the EPHFs can help ensure the health system is prepared to meet the next challenge affordably and sustainably. The findings of the review have been utilized to support high level advocacy for the shift toward public health required to build and ensure health system resilience against future threats. Given the current focus on strengthening public health capacities globally, the findings in Ireland have applicability and relevance to policy audiences and key decision makers within Ireland as well as more broadly to other WHO regions and member states for health systems recovery and building back better, fairer and more resilient health systems.”

The authors apologize for this error and state that this does not change the scientific conclusions of the article in any way. The original article has been updated.

