# Supplementary material for: A novel approach to utilizing the essential public health functions in Ireland's health system recovery and reform
Source: Front Public Health. 2023 Mar 3;11:1074356. doi: 10.3389/fpubh.2023.1074356 (PMC10020328; doi:10.3389/fpubh.2023.1074356)
Supplement: Supplementary file 1 [file Data_Sheet_1.docx]

**Supplementary Information**

**Document Search**

A list of recent (2019) Irish strategy and planning documents that referred to one or more of the EPHFs was compiled using the illustrative list in Table S1. Where recent strategies and policies in key areas were not available but older documents existed, the older documents were included in the review. Ultimately 42 strategy, policy and planning documents were included in the key document review.

**Table S1: Types of documents reviewed**

| **Health systems** |
| --- |
| National Health Sector Strategic Plan and complementary national documents pertaining to national legislation, policy or regulation (e.g. public health legislation, International Health Regulations, 2005 strategic plans) |
| National quality policy or strategy |
| Health system assessments |
| Public health system assessments |
| Health information management system documentation |
| Health financing documentation (e.g. health budget or national health accounts) |
| Health research |
| Subnational operational planning efforts |
| Health workforce strategies with relevance to public health |
| Public health reviews |
| Relevant programme-specific plans and reports (e.g. antimicrobial resistance, immunization and noncommunicable diseases) |
| **Health security** |
| Emergency preparedness and response plans |
| Performance of veterinary services reports |

**Literature Searches to inform EPHF mapping**

Peer reviewed literature search

A review of peer-reviewed and grey literature was undertaken to inform the mapping of EPHFs in the Irish context. Grey literature included both published and non-published materials as identified by key informants from within the Department of Health and the Health Service Executive. Ultimately 24 documents and data from one presentation were included in the review.

Databases*:* PubMed, OVID, Web of Science, EBSCOhost (including Academic Search Complete Business Source Complete, SocINDEX with full text, EconLit and MEDLINE), Scopus and ProQuest (including IBSS Online and ASSIA).

Restrictions: Published December 2011–January 2022; English language only.

Grey literature search

Sites searched: Google, Health Service Executive, Ireland and Department of Health websites, WHO website and four grey literature databases (WHO Iris repository, NBER, Global Health, Open Grey and HISA)

Restrictions: 2011–2021; English language only.

**Inclusion/exclusion criteria**

Included:

- Publications related to one or more EPHF
- Publications from the Irish context
  - Those from countries of similar socioeconomic status (e.g. United Kingdom of Great Britain and Northern Ireland, France, Canada, Germany, and Greece) were also included if of relevance
- Publications at the national or subnational level
- Publications based on empirical data and analysis.

Excluded:

- Publications primarily focusing on clinical care and individual health services
- Publications from middle-/low-/lower-middle-income countries
- Publications that are primarily conceptual and not based on empirical data.

**Table S2: Key Question Matrix**

| **Area** | **Technical questions** | **Cross-cutting questions** |
| --- | --- | --- |
| Policy and planning | - What legislative frameworks support delivery of the EPHFs in terms of individual public health functions (e.g. Ministry of Health, data protection and sharing), and integration between functions and across government departments and sectors as appropriate? - What are the key policies and strategies that support the EPHFs in Ireland?   - Is there evidence of effective implementation of identified strategies?   - What policies govern the mobilization of and allocation of resources for the EPHFs? - How are essential public health services designed and prioritized? - How are the EPHFs incorporated into broader national health sector planning and aligned in terms of content, resources and governance mechanisms? - How is monitoring and evaluation incorporated into policies and planning for the EPHFs (e.g. responsibility, timing and actions}? | What are the strengths, areas for improvement and opportunities in terms of:   - policies, plans and strategies - legislation - infrastructure - service provision and - coordination and integration?   Are there examples of good practice and/or lessons learnt from recent public health events or health system stressors, e.g. COVID-19, that can inform future directions and improvement?  How is monitoring and evaluation of the EPHFs integrated or aligned to support unified goals within and out with the health sector?  Is there evidence of systematic capture and translation of lessons learnt? |
| Inputs and infrastructure | - What are the inputs and infrastructure in Ireland that support delivery of the EPHFs?   - Institutes, laboratories, national and regional units, public health schools, research facilities and clinical care facilities.   - Health workforce (e.g. public health workforce, primary health care workforce and health care workers trained/oriented in public health)     - Is the appropriate skills mix in place to enable delivery of all EPHFs?     - Are skills utilized appropriately, i.e. matched to task?   - Health information systems (e.g. interoperability, mechanisms that promote access to and sharing of surveillance, health service, clinical, demographic and other data for public health with all relevant partners) and other information and communications technology structures   - Supply chains for essential medicines, diagnostics, therapeutics, vaccines and personal protective equipment   - Relevant organizations beyond the health sector - What mechanisms are in place that support learning within the EPHFS? - To what extent is the current infrastructure fit for purpose to meet the current and emerging threats to public health in Ireland? |  |
| Service provision | 1. How are systems and services oriented to deliver and maintain the EPHFs in all contexts? 2. How do the EPHFs affect and influence service provision within Ireland?    1. What mechanisms are in place that enable public health to inform planning, prioritization and resource allocation? 3. What are the processes in place to determine resource allocation in all contexts? 4. Are all EPHFs delivered within current structures, either directly or through defined collaborative partnerships (e.g. research and development, academia and Irish Engineer Corps)? 5. How are preventative and health promotive interventions incorporated into service provision in all contexts? |  |
| Coordination and integration | 1. Who are the key stakeholders involved in the provision of the EPHFs?    1. Within and out with the health sector (e.g. animal, agricultural and environmental actors, community health, army engineer corps, services for vulnerable populations, laboratory services, primary care and the private sector) 2. To what extent are the roles, responsibilities and governance structures of each key stakeholder group clearly defined? 3. What are the mechanisms that support the coordination and integration of the EPHFs in Ireland within the health sector, across sectors, in government departments and at the community level? 4. Is there evidence of duplication of delivery of the EPHFs across the system? 5. What mechanisms are in place that enable a whole-of-government/ whole-of-society approach? 6. Are there examples from recent public health events or health systems stressors such as COVID-19 or economic downturns where a whole-of-government/ whole-of-society approach was used? |  |

**Table S3: Stakeholder Mapping Exercise**

| **Essential public health function** | **Domain(s)** | **Main organization(s)** |
| --- | --- | --- |
| Monitoring and evaluating population health status, health service utilization and surveillance of risk factors and threats to health | Health intelligence | Health Intelligence Unit |
|  |  | Quality and Patient Safety |
|  |  | Health Protection Surveillance Centre |
|  |  | Department of Agriculture, food and the Marine |
|  |  | Regional departments of public health |
|  |  | Central Statistics Office |
|  |  | Hospital groups |
| Public health emergency management | Health protection | National Emergency Coordination Group |
|  |  | National Public Health Emergency Team (CMO/Department of Health) |
|  |  | National and area crisis management teams (interagency) |
|  |  | PICT |
|  |  | CPHOG |
|  |  | National Public Health Emergency Preparedness Group |
|  |  | Port Health Group |
|  |  | Irish Army Engineer Corps |
| Assuring effective public health governance, regulation and legislation | All domains of practice | Department of Health |
|  |  | Health Information and Quality Authority |
| Supporting efficient and effective health systems and multisectoral planning, financing and management for population health | Health service improvement, health protection, health improvement | Health Services Executive, Ireland’s Strategy and Planning |
|  |  | Health Service Improvement Crowe Horwath Group |
| Protecting populations against various and cross-sectoral natural, human-induced and environmental health threats, which may or may not evolve as public health emergencies | Health protection, health service improvement | Health Protection Surveillance Centre |
|  |  | Environmental Health |
|  |  | Department of Agriculture, Food and Marine |
|  |  | Irish Water |
|  |  | National Drinking Water Group |
|  |  | National Bathing Water Group |
|  |  | Health and Safety Authority |
|  |  | Food Safety Authority |
|  |  | Irish Aid |
|  |  | Global Health |
| Promoting prevention and early detection of diseases including noncommunicable and communicable diseases | Health service improvement, health improvement and health protection | National screening programmes |
|  |  | Child Health Programme |
|  |  | National Immunization Office |
|  |  | Regional departments of public health |
|  |  | Health Protection Surveillance Centre |
|  |  | Local authorities |
| Promoting health and well-being and action to address the wider determinants of health and inequity | Health improvement, health service improvement, health protection | Healthy Ireland (Department of Health) |
|  |  | Healthy Ireland (Health Services Executive, Ireland) |
|  |  | CHOs |
|  |  | Hospital groups |
|  |  | Local authorities |
|  |  | Regional departments of public health |
| Ensuring community engagement, participation and social mobilization for health and well-being | Health service improvement, health improvement, health protection | Healthy Ireland/Health and Wellbeing |
| Ensuring adequate quantity and quality of the public health workforce | All domains | National Doctors Training and Planning |
| Assuring quality of and access to health services | Health service improvement, health protection | Health Information and Quality Authority |
|  |  | Quality and Patient Safety |
| Advancing public health research | All domains | Health Information and Quality Authority |
|  |  | Health Services Executive, Ireland, Research and Development |
|  |  | Academic departments |
|  |  | Regional departments |
|  |  | Health Research Board |
| Ensuring equitable access to and rational use of essential medicines and other health technologies |  | Health Information and Quality Authority |

**Table S4: Summary of key public health stressors and challenges in Ireland**

| **Demography and socioeconomic conditions** | Population growth expected over next two decades |
| --- | --- |
|  | Increasing elderly population |
|  | Increasing migrant communities |
|  | Increasing socioeconomic inequity |
| **Population disease profile** | Multimorbidities are the norm rather than the exception |
|  | Increasing rates of obesity |
|  | High rates of cancer, heart disease and respiratory disease |
|  | Increasing mental health issues and substance abuse |
|  | Longer-term impact of COVID-19 |
| **Health systems and infrastructure** | Limited health information systems and cyber security issues |
|  | Health workforce shortages and dissatisfaction |
|  | Primary care and hospital capacity issues |
|  | COVID-19-related disruptions across the health system and services |
| **Population-based health services** | Limited legislation and underinvestment in public health |
|  | Emergency and reactive focus of public health services |
|  | Limited formal linkages to clinical and multisectoral services |

**Table S5: International lessons derived from global experience with COVID-19 with relevance to Irish context**

| **International lessons identified** | **Relevance to the Irish Context** |
| --- | --- |
| Health system resilience is a key enabler of effective emergency response | The COVID-19 pandemic highlighted:   - Weaknesses in acute hospital, ICU and isolation capacity - Underdevelopment of public health infrastructure - Gaps in health information and surveillance - Reliance on private sector purchasing |
| Most countries were unable to effectively deliver the EPHFs due to longstanding lack of proportionate focus and investment in public health capacities | Public health resourcing and capacity were identified as a risk early in the pandemic.   - Spending on prevention in Ireland is lower that EU averages - Much of the initial and sustained investment in response to COVID-19 has remained skewed towards the acute sector |
| Countries who incorporated lessons learned from previous public health events to support health systems appeared more resilient to COVID-19 | Lessons captured following Ireland’s experience with H1N1 in 2009 were not implemented including:   - Need to clarify roles and responsibility of those involved in emergency response - Need to enhance surveillance capacity and develop modelling capacity - Need to review national pandemic preparedness plan |
| Leadership and governance are fundamental to facilitate effective, timely and coordinated national response | A number of issues were highlighted by the pandemic:   - Lack of mandate for monitoring high risk settings - Lack of a legislative basis for International Health Regulations - Lack of emergency response specific legislation - need for substantive amount of (mostly temporary) primary legislation to be drafted - Lack of role definition and clarity in relation to delivery of EPHFs |
| Many countries had weak data and information capacities in relation to health status and health service and surveillance systems. | Ireland delivered high quality data to support decision making through the pandemic. However, numerous weaknesses needed to be overcome:   - Siloed health information with a lack of integration between systems - Procurement of new systems and development and expansion of new data partnerships was needed - Resource intensive workarounds needed, with implications for sustainability |
| A comprehensive and effective public health response requires effective communication | Strong national communication led to a strong sense of solidarity and high level of acceptance of public health measures, including vaccination.   - Clear consistent engagement with media and the public - Behavioral analysis, mobility trends, focused research and testing of planned communication maximized clarity and effectiveness - A Government Information System (GIS) ensured coordination of cross-governmental messaging |
| Public health science, knowledge, research and innovations play a fundamental role in delivering many public health functions | Ireland’s response to the pandemic was evidence-driven.   - COVID-19 Expert Advisory Group was established - The HIQA Evidence Synthesis Team providing rapid, rolling collation and analysis of available evidence to NPHET - Behavioral analysis and mobility trends to support communication - Access to international platforms for experience and expertise sharing - Investment in Innovation and research - e.g. through Health Research Board funding local projects - Innovative approaches to service delivery - e.g. COVID-19 Schools team - a collaboration between public health and The Department of Education |
| Defining and developing the public health workforce and core competencies for public health expertise is essential to build and sustain health system resilience | Ireland employed multiple innovative approaches to expand workforce in public health and acute services   - Bringing forward end of training examinations for final year medical students - Employing student nurses and healthcare attendants - Use of medical retirees - Ireland’s call - international recruitment campaign asking people to register their interest and availability   Despite this there was significant and sustained pressure on the public health workforce. |
| Effective pandemic response requires whole-of-government, whole-of-society planning, approaches, and actions that coordinate efforts across health system functions and levels (national to local) as well as across sectors and society | - Pre-existing structures were leveraged to enable a quicker multi-sectoral response - Joint HSE/DOH pandemic plan was last updated in 2007 - health sector and influenza focused - HPSC pandemic preparedness plan also focused on influenza, developed by the Expert Advisory Group for pandemic flu in 2008 - development of a new plan had commenced in 2019 - paused with the onset of COVID-19 pandemic |
| Community engagement was of paramount importance in fighting COVID-19 | Ireland had a whole-of-society response with evidence of significant levels of community engagement.   - High levels of adherence to public health guidance - Mobilization of individuals and community groups and networks - Leveraging of pre-existing infrastructure to support vulnerable groups |
